# Age-Related Macular Degeneration and Diabetic Retinopathy

**DOI:** 10.3390/jpm12040581

**Published:** 2022-04-05

**Authors:** Andreas Ebneter, Peter D. Westenskow

**Affiliations:** Roche Pharma Research and Early Development, F. Hoffmann-La Roche Ltd., 4070 Basel, Switzerland; andreas.ebneter@roche.com

More than 15 years ago, the results of the pivotal trials supporting the intravitreal use of ranibizumab were published [1,2]. The intravitreal application of anti-vascular endothelial growth factor (anti-VEGF) drugs has subsequently revolutionized the treatment of several common retinal diseases [3]. Suddenly, widespread conditions such as neovascular age-related macular degeneration have become treatable, whereas previously, physicians could only observe and document the rapid deterioration of vision due to irreversible structural retinal damage.

This important paradigm shift in treatment has been further driven by the simultaneous emergence of novel, powerful, non-invasive imaging capabilities. Indeed, the advent of anti-VEGF and optical coherence tomography (OCT) at roughly the same time has advanced retinal treatment in unprecedented ways. OCT has evolved rapidly due to incredible technological advances that have led to a dramatic increase in resolution and acquisition speed. Combined with ingenious software, this has led to the advent of OCT-Angiography, which has the potential to replace the more invasive fluorescein angiography in the not-too-distant future.

In terms of intravitreal treatments, we are now at an inflection point with new treatment modalities emerging. While treatment decisions for intravitreal medications are currently limited to choosing between anti-VEGF and corticosteroids, the need for informed decision making by physicians and choosing the best drug for an individual patient will likely complicate the lives of retina specialists but, hopefully, add more value for patients. We now need to start building the tools to tailor treatment decisions for every individual patient.

For this issue, we approached world-class experts in the fields of medical retina, ocular imaging, and drug development. We have assembled a collection of valuable articles that contain innovative ideas and suggestions for future individualized treatment decisions. We hope you enjoy browsing and reading the articles and that you will be inspired to contribute further and ultimately feel empowered to advance the way we treat patients.

## 1. Imaging Biomarkers

The first section of papers focuses on imaging biomarkers. With dramatic progress in technology for OCT and fundus imaging, we anticipate that more sophisticated data analysis will lead to more tailored treatments for patients and better outcomes.

Sen et al. provide a comprehensive overview of established imaging biomarkers for predicting visual outcomes in diabetic macular edema (DME), which is an important cause of vision loss secondary to diabetes mellitus [4].

Babluch et al. report the 2-year results from the RECOVERY Study in proliferative diabetic retinopathy using an innovative automatic segmentation algorithm to quantify leakage and microaneurysm count, which have been shown to decrease under intravitreal aflibercept treatment. The results suggest that these biomarkers reflect changes in diabetic retinopathy severity score (DRSS) and central subfield thickness well [5].

The 2-year outcomes from the PRIME trial confirm that leakage on ultra-widefield fluorescein angiography can indeed guide intravitreal aflibercept treatment for non-proliferative diabetic retinopathy and help provide individualized treatments to patients. Of note, change in peripheral leakage preceded DRSS worsening [6].

Gadde et al. conducted a very comprehensive volumetric analysis on spectral-domain OCT in patients with diabetic retinopathy. The edema volume correlated with diabetic retinopathy severity and predicted the response to intravitreal treatment. Edema volume increased with disease severity and was the best predictor for response to treatment [7].

To reflect the current state of development, the review by Kalra et al. provides a comprehensive review of currently used quantitative imaging biomarkers in age-related macular degeneration (AMD) and diabetic eye disease. Various approaches to extracting the pertinent information are also presented [8].

The findings by Lai et al. illustrate that morphological aspects can be directly related to function. This real-world data study confirms previous reports from clinical trials that greater fluctuations in retinal thickness are associated with worse visual outcomes [9].

## 2. Systemic Biomarkers

Existing evidence suggests that glucose variability might be an independent risk factor for the development of diabetic retinopathy. However, quantification of glucose variability is costly. Hsing et al. investigated the possibility of using the glycemic gap as a surrogate for glucose variability. Findings suggest that a negative glycemic gap is associated with progression and show the importance of minimizing glucose fluctuations [10].

Prasuhan et al. explored the predictive value of specific autoantibodies in neovascular AMD. Unfortunately, the five autoantibodies investigated were not useful in predicting the clinical course. However, the selection was limited, and the outcome highlights the importance of a more holistic approach [11].

In addition, Vader et al. reported results of a study designed to determine if circulating microRNAs (miRs) levels could predict patient responsiveness to anti-VEGF therapies in patients with DME. Their findings suggest that miR-181a may be negatively associated with the central area thickness of the retina at baseline. Work focused on miRs is emerging, and it is exciting to think similar trends can be determined as we identify more systemic biomarker candidates [12].

Finally, Acar et al. report that multiplex profiling of complement proteins, demographic, factors, genetic variants, and systemic metabolites are correlated in patients with AMD. This holistic approach is exciting as it may ultimately provide clinicians with more options when treating patients [13].

## 3. Public Health Aspects

In the paper by Nugawela et al., a different angle is taken to highlight the importance of ethnic and socio-economic aspects in diabetic eye disease care. The group investigated the influence of ethnicity on the risk of DR and sight-threatening complications in the UK. The outcomes show the importance of improving prevention, screening and treatment for specific ethnic minorities [14].

## 4. Innovative Technologies

This section of this Special Issue focuses more on technical aspects and innovative technology.

Maunz et al. show that their machine learning/artificial intelligence approach based on spectral-domain OCT images from the HARBOR trial was accurate in classifying choroidal neovascular lesions in exudative AMD. Results highlight the power of machine learning and suggest that the different OCT modalities have the potential to substitute fluorescein angiography in the very near future [15].

Cunha-Vaz et al., in their pilot study, experimented with an advanced OCT-Angiography-based grading system to substitute DRSS grading on color fundus images [16].

Frizziero et al. report interesting results from a subthreshold micropulse laser treatment for diabetic macular edema. Different groups have used this innovative method to treat macular edema, but its role is still not entirely clear. These results suggest that the subthreshold micropulse laser is safe and repeatable for mild diabetic macular edema. Such treatment might be considered for stabilizing macular edema in settings where other alternatives are lacking [17].

In addition, Maurissen et al. reviewed the current and future opportunities for modeling neurovascular interactions using organ-on-a-chip and other physiological microsystems. They argue that optimized models of the inner brain–retinal–blood barrier (iBRB) can be generated using the knowledge obtained from studies of the neurovascular unit in the retina and brain. Developing robust iBRB models could allow vision researchers to test key hypotheses about iBRB integrity and identify novel targets to prevent devastating features of diabetic retinopathy and other neurovascular diseases [18].

## Data Availability

Not applicable.
